# Supplementary material for: Heterogeneous expression of the SARS-Coronavirus-2 receptor ACE2 in the human respiratory tract
Source: eBioMedicine. 2020 Sep 21;60:102976. doi: 10.1016/j.ebiom.2020.102976 (PMC7505653; doi:10.1016/j.ebiom.2020.102976)
Supplement: Supplementary file 1 [file mmc1.doc]

**Supplemental information:**

**Supplemental Figure 1**


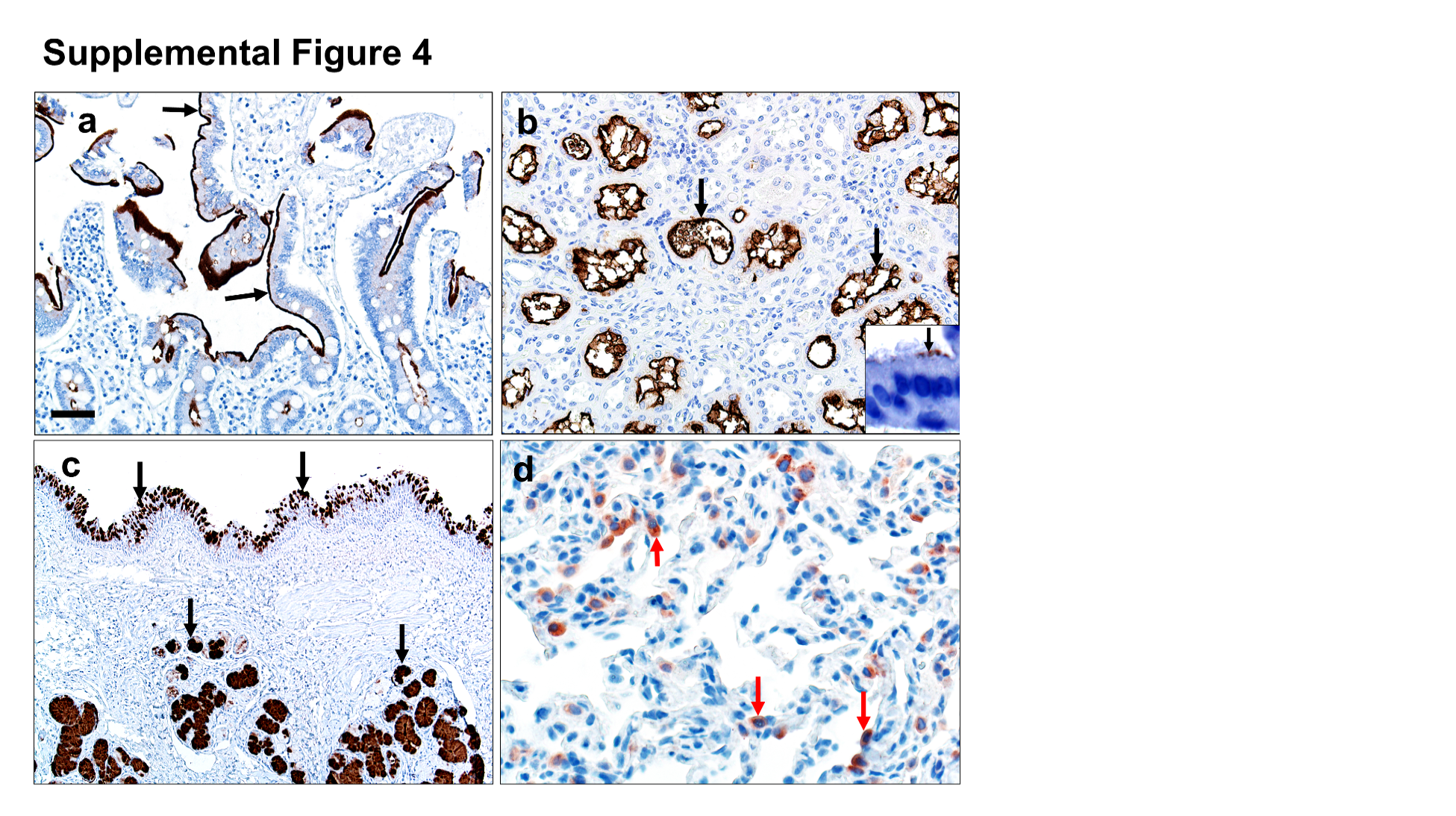


Quality controls for ACE2 immunohistochemistry technique (**a, b**) and tissue quality (**c, d**). **a, b**) ACE2 protein (brown color, black arrows) was detected along the apical surface of small intestine enterocytes (**a**), renal tubule epithelium (**b**), and ciliated cells (**b, inset**) of primary airway cell cultures. These findings demonstrate specific detection of ACE2 protein in cells/tissues consistent with known ACE2 expression. **c**) Representative immunostaining of bronchus detected abundant MUC5B protein (brown color, black arrows) in mucous cells of surface epithelium (top) and submucosal glands (bottom). **d**) Representative sections of alveoli had SP-C^+^ alveolar type II cells (red color, red arrows). These results (**c, d**) demonstrate the tissues were intact and that immunostaining can be used to detect native airway (**c**) and lung (**d**) proteins. Bar = 40 (a, b), 80 (c), and 20 μm (d).

**Supplemental Figure 2**

**
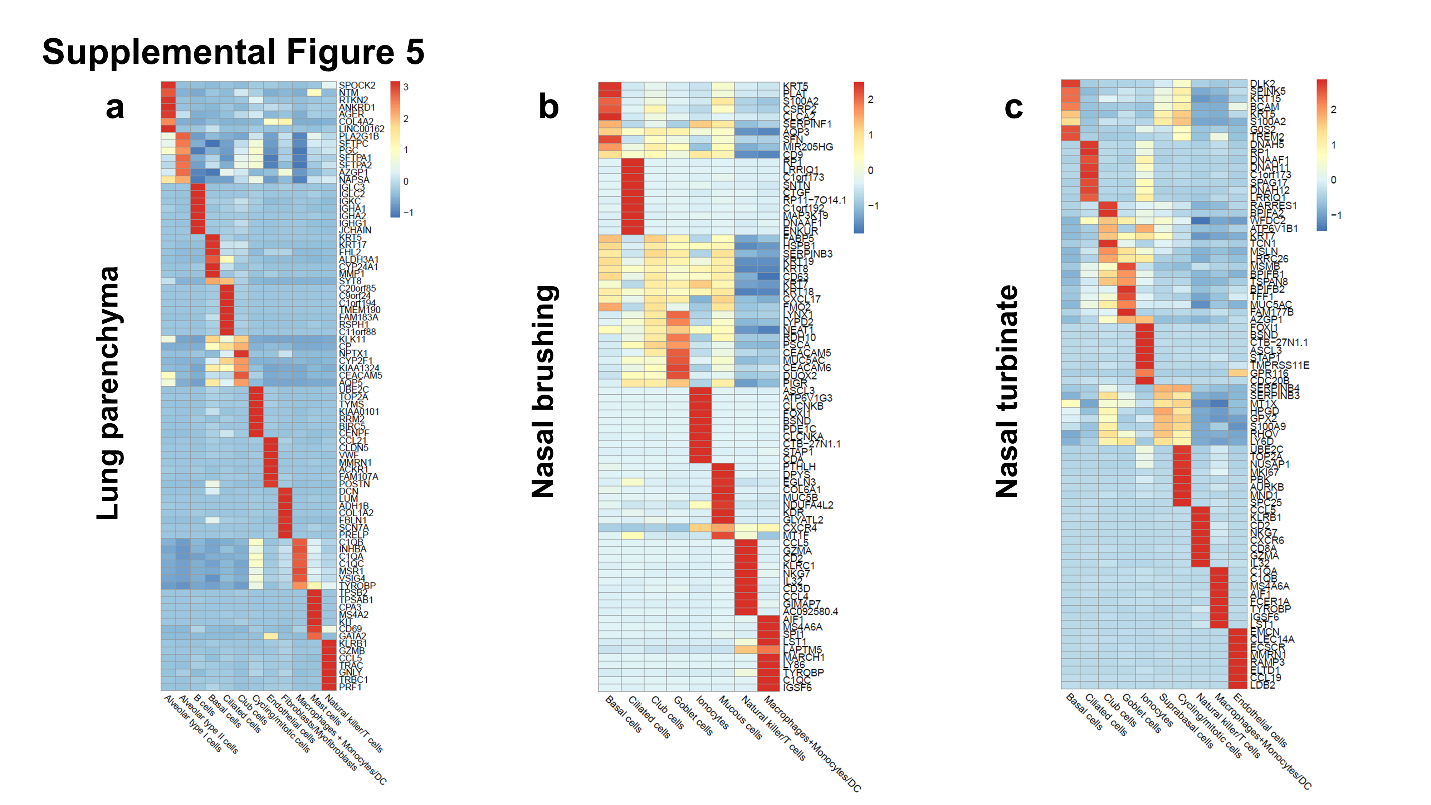
**

Single-cell RNA sequencing reanalyses of lung parenchyma (a) ^1^, nasal brushing (**b**), and nasal turbinate (**c**) ^2^. Heatmaps depicting the marker genes for each cluster that were used to assign cell types.

**Supplemental Figure 3**

**
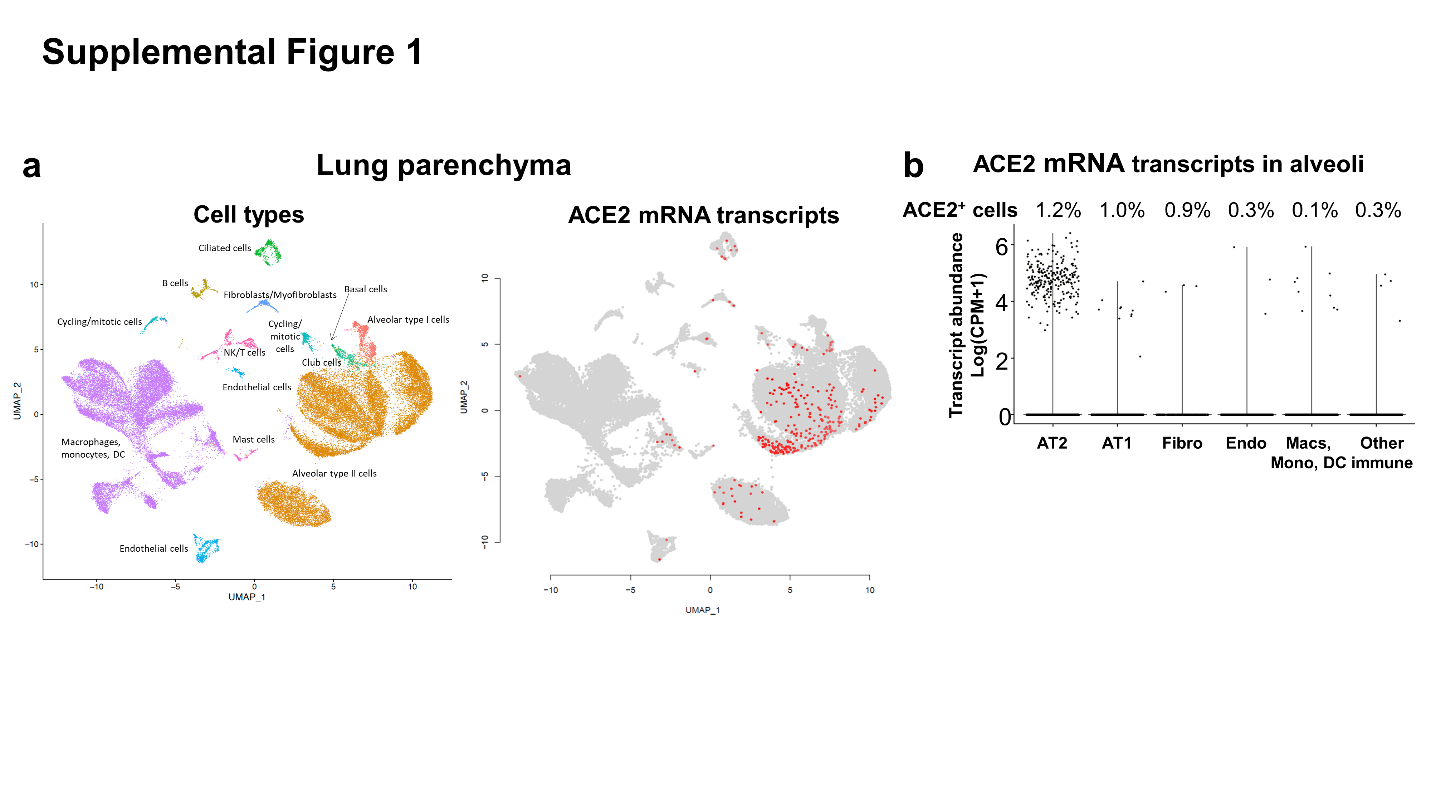
**

Single-cell RNA sequencing reanalyses of ACE2 transcript abundance in lung parenchyma ^1^. Summative observations from all donors. **a**) Uniform manifold approximation and projection (UMAP) visualizations. Cells were clustered using a shared nearest neighbor (SNN) approach. Cell types associated with each cluster were identified by determining marker genes for each cluster. Each data point denotes a cell. On the right panel, cells with ACE2 transcripts are shown in red. **b**) Violin plots representing ACE2 expression in the alveoli. Airway cells (basal, mitotic, ciliated, club) are not shown. Percentage of ACE2^+^ cells within each cell type shows ACE2 transcripts in 1·2% of alveolar type II cells and in 0·1% of macrophages, monocytes, or dendritic cells. Each data point denotes a cell, most cells have no expression (0). AT2: alveolar type II. AT1: alveolar type I. Macs: Macrophages. Mono: Monocytes. DC: dendritic cells. Other immune cells: B cells, mast cells, natural killer/T cells. Endo: Endothelial. Fibro: Fibroblasts/myofibroblasts. NK: Natural killer. CPM: Counts per million.

**Supplemental Figure 4**


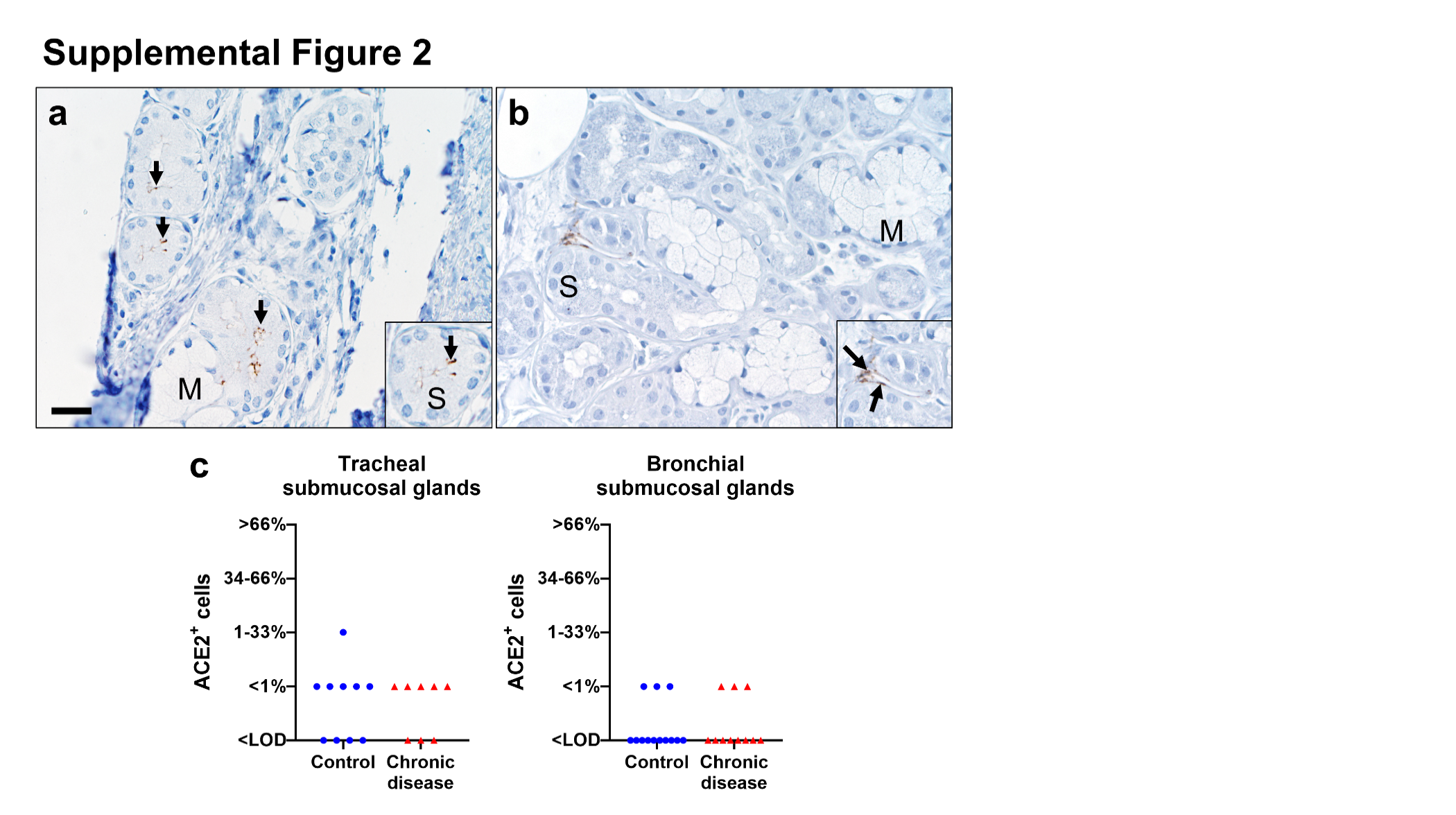


Representative tissue section from submucosa of large airways (trachea/bronchi) showing ACE2 protein localization (brown color, black arrows) (**a, b**) and scores (**c**). **a**) Submucosal glands had uncommon to localized apical ACE2 protein (arrows) in serous (S) cells, but not mucous (M) cells. **b**) Submucosal glands also had absent to uncommon ACE2 protein (arrows) in the interstitium that centered on vascular walls and endothelium. This vascular staining was uncommonly seen in lung too and corresponded to the low levels seen in transcripts for these endothelial cells (Supplemental Figure 3a-b). Note the absence of ACE2 staining in serous (S) or mucous (M) cells of the gland (**b**). **c**) ACE2 protein scores for each subject for serous cells in submucosal glands from trachea and bronchi, in control versus chronic disease groups (P>0·9999, 0·9999, respectively, Mann-Whitney U test). Bar = 25 μm. LOD: Limit of detection.

**Supplemental Figure 5**


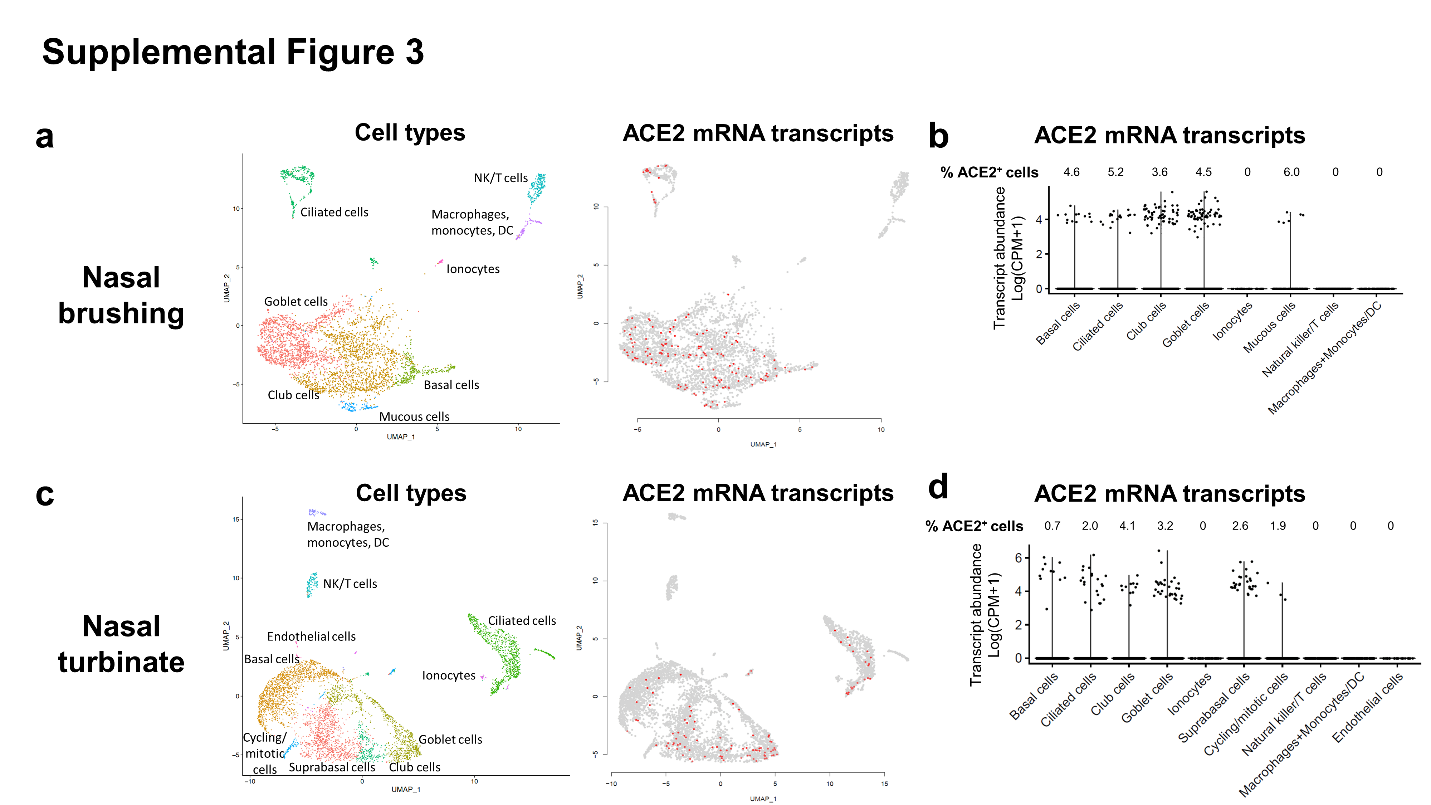


Single-cell RNA sequencing reanalyses of ACE2 transcript abundance in nasal brushing (**a, b**) and nasal turbinate (**c, d**) ^2^. **a, c**) Uniform manifold approximation and projection (UMAP) visualizations. Cells were clustered using a shared nearest neighbor (SNN) approach. Cell types associated with each cluster were identified by determining marker genes for each cluster. Each data point denotes a cell. On the right panels, cells with ACE2 transcripts are shown in red. **b, d**) Violin plots representing ACE2 expression. In nasal turbinate and nasal brushing, percentage of ACE2^+^ cells within each cell type shows ACE2 expression on epithelial cells. Each data point denotes a cell, most cells have no expression (0). DC: dendritic cells. NK: Natural killer. CPM: Counts per million.

**Supplemental Table 1**

**ACE2 protein reported in surface epithelium (SE) of human respiratory tract surface epithelium**

| **Reported Cases (n)** | **Primary Ab** | **SN** | **T** | **B** | **Br** | **Al** | **Summary comments** |
| --- | --- | --- | --- | --- | --- | --- | --- |
| Non-diseased lungs / nasal (5 each); diseased lungs (5) ^3^ | Polyclonal | SE (C++, basal cells in squamous epithelium) | n.d. | SE (C+) | n.d. | AT1 (C++); AT2 (C++) | Abundant ACE2 protein in lung epithelia |
| Non-diseased lungs (5) ^4^ | Undefined | n.d. | SE (C+, A+) | SE (C+, A+) | n.d. | "Alveoli" (A+)  Mac (A+) | ACE2 is present on epithelia in several parts of the respiratory tract and macrophages |
| Lung (undefined) ^5^ | Polyclonal | n.d. | n.d. | SE (C+, N+, M+) | n.d. | AT1 ̶  AT2 (N+) | ACE2 is present in bronchial epithelium, AT2 cells, and macrophages |
| Sinus (undefined) and Lung (undefined, same tissues as above) ^6^ | Polyclonal | SE (N++) | SE ( ̶ ) | SE (C+, N++) | n.d. | AT1 ̶  AT2 (N++) | ACE2 is present in sinus and bronchial epithelium, AT2 cells, and macrophages |

Non-diseased: The cause of death was not directly related to lung disease

n.d.: Not described

Tissues: Sinonasal (SN), trachea (T), bronchi (B), bronchioles (Br), and alveoli (Al)

Cellular localization: cytoplasmic (C), nuclear (N), apical membrane (A)

Cells: Surface epithelium (SE), alveolar type I cells (AT1), alveolar type II cells (AT2), alveolar macrophages (Mac)

ACE2 protein (based on published reports/figures): negative ( ̶ ), weak (+), moderate to abundant (++)

**Supplemental Table 2**

**Donor demographics and ACE2 distribution scores for each tissue region**

| **Case #** | **Group** | **Age (yrs)** | **Sex** | **Comorbidities** | **Trachea** | **Bronchi** | **Bronchioles** | **Alveoli** |
| --- | --- | --- | --- | --- | --- | --- | --- | --- |
| 1 | Control | 5 | F | Trauma | NA | 2 | 2 | 1 |
| 2 | Control | 57 | M | Arrhythmia | 0 | 0 | 0 | 1 |
| 3 | Control | 31 | M | Stroke (Joubert syndrome) | 1 | 1 | 0 | 0 |
| 4 | Control | 53 | F | Trauma | NA | 0 | 0 | 1 |
| 5 | Control | 2 | M | Brain hemorrhage | 0 | 0 | 0 | 1 |
| 6 | Control | 2 | M | Trauma | 0 | 0 | 1 | 2 |
| 7 | Control | 0·5 | M | Spinomuscular atrophy | NA | 0 | 1 | 0 |
| 8 | Control | 71 | M | Stroke, Parkinson's disease, nonsmoker | 0 | 1 | 1 | 0 |
| 9 | Control | 4 | F | Trauma | 0 | 0 | 0 | 2 |
| 10 | Control | 1·2 | M | Trauma | 0 | NA | 1 | 1 |
| 11 | Control | 53 | F | Trauma, nonsmoker | 0 | 0 | 2 | 0 |
| 12 | Control | 26 | F | NA | 0 | NA | 0 | 0 |
| 13 | Control | 27 | F | NA | NA | 0 | 1 | 0 |
| 14 | Control | 64 | M | NA | NA | 1 | 1 | 0 |
| 15 | Chronic disease | 53 | F | Smoker | 0 | NA | 0 | 1 |
| 16 | Chronic disease | 60 | M | COPD, smoker | NA | NA | 0 | 1 |
| 17 | Chronic disease | 32 | M | COPD, smoker | 0 | 0 | 0 | 1 |
| 18 | Chronic disease | 68 | M | COPD | NA | 1 | 0 | 1 |
| 19 | Chronic disease | 68 | F | COPD | NA | NA | 1 | 1 |
| 20 | Chronic disease | 9 | M | Asthma | 0 | 0 | 0 | 1 |
| 21 | Chronic disease | 25 | F | Cystic fibrosis | NA | 0 | 0 | 0 |
| 22 | Chronic disease | 47 | F | Cardiovascular disease | 1 | 2 | 2 | 1 |
| 23 | Chronic disease | 27 | M | Cystic fibrosis | 0 | NA | NA | 1 |
| 24 | Chronic disease | 50 | F | Cardiovascular disease, diabetes, asthma | NA | 0 | 0 | 0 |
| 25 | Chronic disease | 37 | M | Drug use, smoker | 0 | 0 | 0 | 0 |
| 26 | Chronic disease | 38 | M | Asthma (status asthmaticus) | 0 | 0 | 0 | 0 |
| 27 | Chronic disease | 32 | M | Cystic fibrosis | NA | NA | 0 | 1 |
| 28 | Chronic disease | 58 | F | Cardiovascular disease, diabetes, NASH | 0 | 0 | 0 | 1 |
| 29 | Chronic disease | 19 | F | Cystic fibrosis | NA | 0 | 0 | 0 |

NA: Not available for analyses / COPD: Chronic obstructive pulmonary disease / NASH: Non-alcoholic steatohepatitis.

Scoring: 0 = below limit of immunohistochemical detection; 1 = rare (<1%); 2 = 1-33%; 3 = 34-66%; 4 = >66% of cells.

**Supplemental Table 3**

**Parameters for immunohistochemistry on fixed tissues**

| **Target** | **Primary Antibody** | **Antigen Retrieval** | **Secondary Reagents** |
| --- | --- | --- | --- |
| Angiotensin-Converting Enzyme 2 (ACE2) | Anti-ACE2, monoclonal (MAB933, R&D Systems, Minneapolis, MN USA) in diluent at 1:100 x 1 hour. | HIER, Citrate Buffer, pH 6·0, 110˚C for 15 minutes; 20 min cool down (Decloaking Chamber Plus, Biocare Medical, Concord, CA USA) | Dako EnVision+ System- HRP Labeled Polymer Anti-mouse, 60 min (Dako North America, Inc., Carpentaria, CA USA), DAB Chromogen, counterstain. |
| MUC5B | Rabbit anti-MUC5B polyclonal, (LSBio #LS-B8121, LifeSpan BioSciences, Inc., Seattle, WA) in Dako Antibody Diluent (Dako North America, Inc., Carpentaria, CA); 1:60,0000/30 min | HIER, Citrate buffer pH 6·0, 110˚C for 15min; 20 min cool down | Step 1: Biotinylated anti-Rabbit IgG (H+L) (Vector Laboratories, Inc., Burlingame, CA) in Dako Wash Buffer (Dako North America, Inc., Carpentaria, CA); 1:500, 30 min  Step 2: Vectastain ABC Kit (Vector Laboratories, Inc., Burlingame, CA), 30min. DAB Chromogen, counterstain. |
| Surfactant Protein–C (SP-C) | Anti-SP-C, polyclonal (PA5-71680, Thermo Fisher Scientific, Waltham, MA USA) in diluent 1:100 x 1 hour | HIER, Citrate Buffer, pH 6·0, 110˚C for 15 minutes; 20 min cool down (Decloaking Chamber Plus, Biocare Medical, Concord, CA USA) | Dako EnVision+ System- HRP Labeled Polymer Anti-rabbit, 60 min (Dako North America, Inc., Carpentaria, CA USA), AEC chromogen, counterstain. |

HIER – Heat-induced epitope retrieval

DAB – 3,3'-Diaminobenzidine (produces brown stain)

AEC - aminoethyl carbazole (produces red stain)

Counterstain – Harris hematoxylin (blue color)

**References for supplemental information:**

1. Reyfman PA, Walter JM, Joshi N, et al. Single-Cell Transcriptomic Analysis of Human Lung Provides Insights into the Pathobiology of Pulmonary Fibrosis. *Am J Respir Crit Care Med* 2019; **199**(12): 1517-36.

2. Ruiz Garcia S, Deprez M, Lebrigand K, et al. Novel dynamics of human mucociliary differentiation revealed by single-cell RNA sequencing of nasal epithelial cultures. *Development* 2019; **146**(20).

3. Hamming I, Timens W, Bulthuis ML, Lely AT, Navis G, van Goor H. Tissue distribution of ACE2 protein, the functional receptor for SARS coronavirus. A first step in understanding SARS pathogenesis. *J Pathol* 2004; **203**(2): 631-7.

4. Ren X, Glende J, Al-Falah M, et al. Analysis of ACE2 in polarized epithelial cells: surface expression and function as receptor for severe acute respiratory syndrome-associated coronavirus. *J Gen Virol* 2006; **87**(Pt 6): 1691-5.

5. Bertram S, Glowacka I, Muller MA, et al. Cleavage and activation of the severe acute respiratory syndrome coronavirus spike protein by human airway trypsin-like protease. *J Virol* 2011; **85**(24): 13363-72.

6. Bertram S, Heurich A, Lavender H, et al. Influenza and SARS-coronavirus activating proteases TMPRSS2 and HAT are expressed at multiple sites in human respiratory and gastrointestinal tracts. *PLoS One* 2012; **7**(4): e35876.
